# Investigation of Microstructure, Oxides, Cracks, and Mechanical Properties of Ti-4Al-2V Joints Prepared Using Underwater Wet Laser Welding

**DOI:** 10.3390/ma17081778

**Published:** 2024-04-12

**Authors:** Yonghui Zhu, Yujia Zhang, Congwei Li, Jialei Zhu, Lu Wang, Chao Fu

**Affiliations:** 1 Nuclear Power Institute of China, Chengdu 610213, China; 13548013148@163.com (Y.Z.); damattochang@163.com (Y.Z.); 2 Tianjin Key Laboratory of Advanced Joining Technology, School of Materials Science and Engineering, Tianjin University, Tianjin 300354, China; licongwei1_@tju.edu.cn; 3 School of Mechanical Engineering, Beijing Institute of Petrochemical Technology, Beijing 102617, China; zhujialei@bipt.edu.cn; 4 School of Materials Science and Engineering, Sichuan University, Chengdu 610065, China

**Keywords:** Ti-4Al-2V, underwater wet welding, laser welding, oxide layer, crack

## Abstract

Developing advanced underwater welding technology for titanium, which is the key structural material for underwater applications, is of great significance for the design, fabrication, and maintenance of submarine equipment. In this study, in order to investigate the underwater welding microstructure and mechanical properties of Ti-4Al-2V alloy, underwater wet laser welding was conducted on Ti-4Al-2V alloy using varying laser power. The microstructure and properties of the welding joints were characterized and analyzed. The microstructure of the heat-affected zone and fusion zone in the welding joints are not significantly different from those of welding in air, but a mixed oxide layer composed of Al_2_O_3_ and TiO_2_ is formed on the surface of the fusion zone. Due to internal stress, a large number of cracks initiate on the oxide layer and propagate to the joints. In the 4 kW and 5 kW joints, a penetrating crack formed due to the excessive accumulation of internal stress breaking up the α phase. The mechanical properties of the joints are significantly affected by the laser power. The tensile strength of the 3 kW and 4 kW joints is comparable to that of the base metal, which is about 600 MPa, while the 5 kW joint shows brittle fracture with no plastic deformation and 228 MPa strength. This research lays a solid foundation for understanding the underwater wet laser welding behavior of titanium alloys.

## 1. Introduction

Titanium alloys have a series of advantages, including high specific strength, good corrosion resistance, stable mechanical properties at high and low temperatures, as well as excellent welding and machining performance [1,2,3]. In addition, the excellent resistant to seawater and salt spray corrosion makes titanium alloy a new type of structural material in marine environments. It is widely used in fields such as ships, deep-sea exploration, oil and gas exploitation, aerospace, petrochemicals, and seawater desalination [4,5,6,7]. Countries such as the United States, Russia, and China have been exploring the application of titanium alloys as marine materials since the 1960s. Russia developed the world’s first all-titanium alloy nuclear submarine in 1968. In recent years, with developments in science and technology, the application of titanium alloys in China’s marine industry has gradually increased, among which, the “Jiaolong” deep submarine uses titanium alloy as shells.

Ti-4Al-2V, a near α titanium alloy, has been applied in marine engineering in China and Russia [8]. Previous studies on Ti-4Al-2V alloy mainly focused on its properties of oxidation and irradiation resistance, and formation capability during additive manufacturing and welding in air [9,10,11,12]. However, the research on underwater welding of Ti-4Al-2V alloy is still absent, which is not conducive to its long-term stable service as a key structural material for submarine equipment, and there is no technology guidance for its maintenance.

The history of developing underwater welding technology is long, and the commonly used underwater welding methods include dry welding, local dry welding, and wet welding [13,14,15]. Compared with the first two methods, underwater wet welding has the advantages of low cost and simple operation, and underwater flux-cored arc welding has been widely used [16,17]. Underwater wet welding for titanium alloys, especially the laser welding which is more efficient and easier to achieve with automation, is still in the initial stages. For titanium alloys, most of the research on underwater laser welding is focused on local dry underwater welding [18,19,20], which was conducted in a similar environment to air welding and cannot reflect the particularity of underwater welding. Cai et al. conducted research on underwater wet welding of TC4 alloy, investigating the microstructure and mechanical properties of joints under different water depths [21]. In their study, water depth has a significant impact on the penetration depth of laser welding and has a significant impact on the formation of the joints. Meanwhile, oxidation was observed on the surface of the weld joints, but no detailed analysis was conducted. However, the research on underwater wet laser welding of titanium alloys is scarce, which cannot support the future development and application of Ti-4Al-2V in underwater environments.

In this work, weld joints were prepared with 3 kW, 4 kW, and 5 kW laser power at a water depth of 4 mm, to investigate the underwater welding microstructure, and mechanical properties of Ti-4Al-2V alloy. The morphology, microstructure, texture and variants, and oxidation of the welding joints was characterized and analyzed. The formation and propagation mechanism of cracks during underwater wet laser welding were preliminarily explained. Meanwhile, the tensile properties and micro-Vickers hardness of the joints at room temperature were tested. Based on these, the relationships between laser power, microstructure, and mechanical properties were analyzed. A systematic understanding of the underwater wet welding behavior of Ti-4Al-2V alloy was obtained.

## 2. Materials and Methods

**Figure 1:**
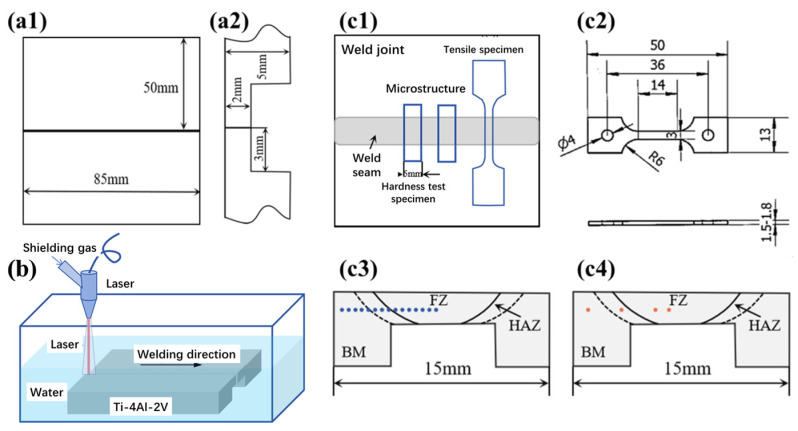
Schematic diagram for (**a1**,**a2**) welding specimens; (**b**) Ti-4Al-2V underwater wet laser welding; schematic diagram of (**c1**) sampling location and (**c2**) sample size for tensile tests; and locations for (**c3**) hardness tests and (**c4**) microstructure characterization.

In this study, a base metal (BM) plate with the nominal composition of Ti-4Al-2V and 85 mm × 50 mm × 5 mm in size was machined to form a butt joint with a thickness of 2 mm. The welding groove form is I-shaped, and the specific dimensions are shown in Figure 1(a1,a2).

The RFL-C6600S (Wuhan Raycus Fiber Laser Technologies Co., Ltd., Wuhan, China) continuous fiber laser was used in the underwater wet laser welding, with a fixed defocus amount of +35 mm and a spot diameter of 5 mm. During the welding process, the joint was formed in one attempt. Figure 1b shows the schematic diagram of the welding process; the upper surface of the specimen was 4 mm away from the water surface. The welding speed was 5 mm/s, the shielding gas flow rate was 50 L/min, and three different laser power settings were used, namely, 3 kW, 4 kW, and 5 kW.

The room temperature tensile properties and micro-Vickers hardness were tested, and the tensile samples’ cutting position was as shown in Figure 1(c1). The microhardness test covered the BM, heat-affected zone (HAZ), and fusion zone (FZ) of the weld joints. The tensile test specimen was of the size shown in Figure 1(c2), and was cut from the joint center without cracking. The tensile tests for each weld joint were repeated two times with a tensile rate of 0.00025 s^−1^, to obtain reliable results. The electronic universal tensile testing machine TSE-OM-20208A (Wance testing machine Co., Ltd., Shenzhen, China) was used to conduct room temperature tensile tests according to GB/T228 [22]. The Vickers hardness on the surface of the welding joints was tested using the FALCOON511 (Innovatest Shanghai Co, Ltd., Shanghai, China) hardness testing machine in accordance with GB/T4340.1 [23], along the path from left to right as shown in Figure 1(c3), covering the BM, HAZ, and FZ. The test used an indenter of 50 g with a holding time of 15 s and a step size of 0.5 mm.

The microstructure of the BM, HAZ, and FZ in the welding joints was investigated using an optical microscope (OM) and a scanning electron microscope (SEM) at the positions shown in Figure 1(c4). The samples were mechanically ground and polished, and etched in a mixed solution of HF:HNO_3_:H_2_O = 1:4:15 (in volume) at room temperature for 15 s. A Phenom XL (Phenom-World, Eindhoven, The Netherlands) scanning electron microscope was used to characterize the weld cracks and surface oxides. The phase composition of the welding joints was characterized using X-ray diffraction (XRD), Cu target, K_α_ Ray, an incident wavelength of 0.154 nm, a diffraction range of 20~90°, and a diffraction speed of 2°/min. The fracture morphology of the tensile specimen was observed using SEM after ultrasonic cleaning in anhydrous ethanol for 5 min.

## 3. Results and Discussion

### 3.1. Macroscopic Morphology

**Figure 2:**
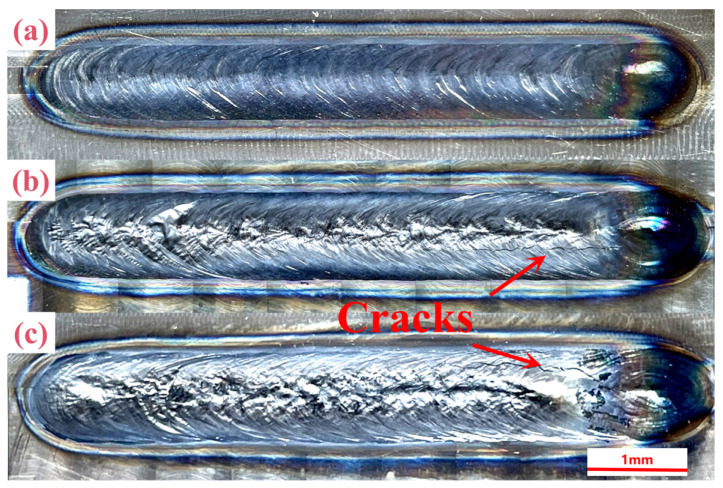
Macroscopic morphology of Ti-4Al-2V weld joints formed via underwater wet welding using different laser power: (**a**) 3 kW, (**b**) 4 kW, (**c**) 5 kW.

Figure 2 shows the macroscopic morphology of the Ti-4Al-2V joints formed via underwater wet welding using different laser powers of 3 kW, 4 kW and 5 kW. The surface of all the three joints is slightly oxidized and in the color of pale blue. The width of welding increases with the increase in laser power, namely, 0.94 ± 0.13 mm, 0.98 ± 0.15 mm, and 1.05 ± 0.18 mm for 3 kW, 4 kW and 5 kW, respectively, which is consistent with general understanding. The surface of the 3 kW joint is smooth, and an obvious fish scale pattern can be seen in Figure 2a, while the surface of the 4 kW and 5 kW joints is uneven, and cracks can be seen at the latter part of the joints as shown by the red arrows in Figure 2b,c, which are almost in line with the welding direction.

**Figure 3:**
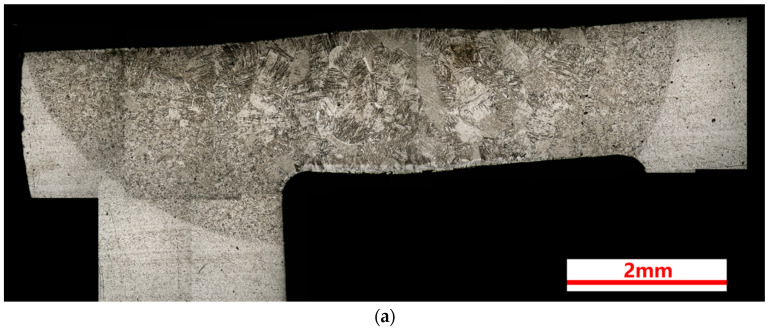

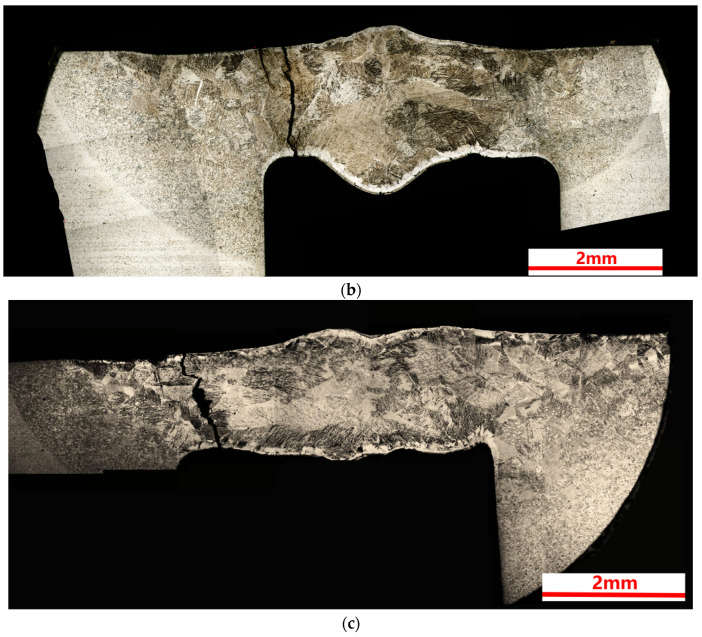
Cross section metallographic structure of Ti-4Al2V joints formed via underwater wet welding using different laser power: (**a**) 3 kW, (**b**) 4 kW, (**c**) 5 kW.

Figure 3 shows the cross-section metallographic morphology of the three welding joints. As shown in Figure 3a–c, a penetration depth of more than 2 mm can be achieved with a laser power of 3 kW. The boundary between the HAZ and the BM is obvious, and it shows a tendency to shrink from both sides to the center from top to bottom. However, the boundary between the FZ and HAZ is not so clear, but it can still be distinguished from the micromorphology; the grain size of the FZ is significantly larger than that of the HAZ. In addition, the columnar grains are the typical morphology in the FZ, as shown in Figure 3, and the grain growth direction at the edge of the FZ is perpendicular to the interface. The grain morphology in the HAZ is between the FZ and the BM, and the grains in the HAZ exhibit a gradient distribution from the FZ to the BM.

A layer of white contrast can be seen at the upper and lower surface of the FZ, which was suspected to be oxide, and its thickness increases significantly with the increase in laser power. The oxide morphology and composition will be further analyzed in Section 3.2. In Figure 3b,c, there is a penetrating crack in the FZ, and some irregular small notches at the edge of the joint. The 4 kW joint also has a non-penetrating crack at the top that extends inward from the upper surface. Crack formation and propagation are further analyzed in Section 3.4.

### 3.2. Microstructure and Oxide Layer

**Figure 4:**
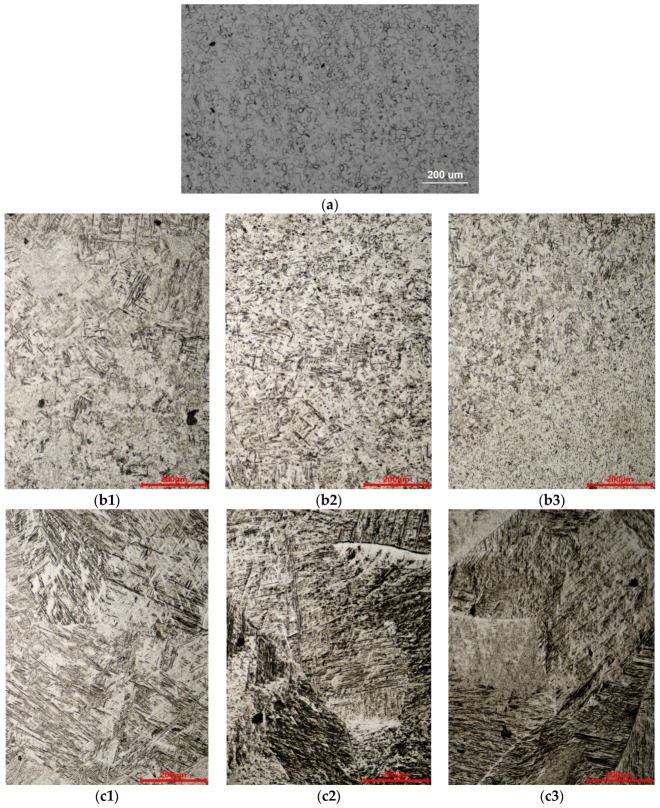
Metallographic images of Ti-4Al-2V joints formed via underwater wet welding using different laser power: (**a**) BM, (**b1**–**b3**) HAZ of 3 kW, 4 kW and 5 kW joints, (**c1**–**c3**) FZ of 3 kW, 4 kW and 5 kW joints.

The microstructure of the BM, HAZ, and FZ was locally magnified, as shown in Figure 4(a–c3). As shown in Figure 4a, the grains in the BM are fine, homogeneous and equiaxed α grains formed by forging and annealing. In Figure 4(b1–b3), the grains in the HAZ are coarser with a slat-like phase inside, which is a martensite phase formed by the α → β → α transformation during heat treatment [24,25]. The grain size in the FZ are the largest with a columnar phase inside, which is related to the temperature gradient of the molten pool, and its priority growth direction is the heat flow direction [4]. The microstructure of the FZ grains is complex, and a needle-like phase can be detected, which is also the martensite phase formed by the α → L → β → α (α′) transformation during melting and rapid cooling [26].

**Figure 5:**
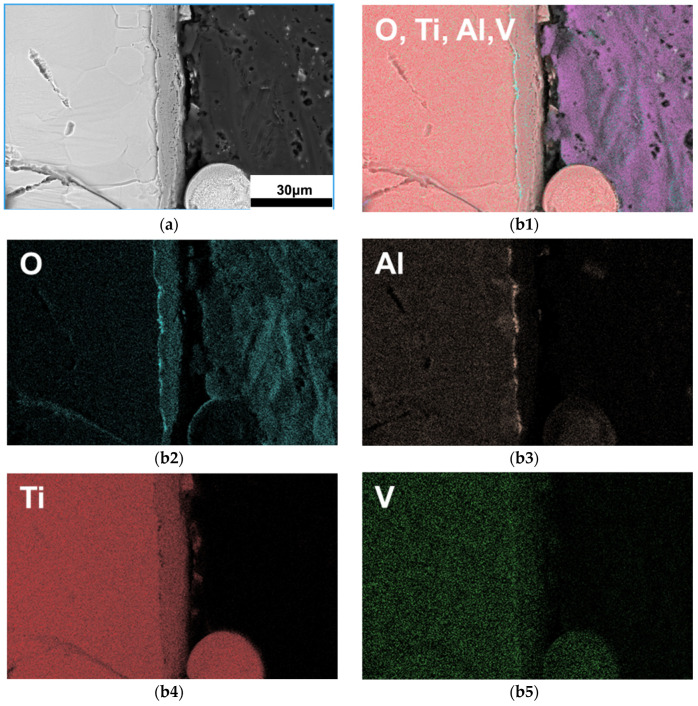
SEM images of surface oxide in the FZ: (**a**) BSD morphology of the FZ, (**b1**–**b5**) EDS mapping of the oxide layer.

In order to further investigate the white contrast phase (suspected as oxide) on the FZ surface, the FZ of the 5 kW joint was characterized using the secondary electron (SE2), back scatter diffraction (BSD), and energy dispersive spectrometer (EDS) mode of the electron scanning microscope. As shown in Figure 5, EDS images proved that the FZ surface is indeed coated by oxide in a thickness of approximately 10 μm. The oxidation degree increases with the laser power reflected by the pale blue color on the surface of the joints shown in Figure 2. The oxide layer is divided into inner and outer layers as shown in Figure 5(b1–b5). The thin inner layer is about 1 μm, enriched with an Al element, which should be Al_2_O_3_, and the outside thicker oxide layer is mainly enriched with a Ti element, which should be TiO_2_.

Oxidation of titanium during underwater welding is inevitable; however, reports on the oxidation of underwater wet welding joints are scarce. Cai et al.’s study also found oxide on the surface of a TC4 alloy after underwater wet laser welding, but a detailed analysis was not conducted [21]. The laser passing through water reaches the surface of the titanium alloy, melts the alloy and releases a large amount of heat at the same time. The water decomposes into H_2_ and O_2_, and O_2_ dissolves into liquid titanium, producing an oxide layer on the surface of the molten pool [27]. As the laser power increases, more heat is generated at the interface of Ti-4Al-2V and water and the oxide film thickness increases, as shown in Figure 5.

### 3.3. Phase Composition and Variant Selection

**Figure 6:**
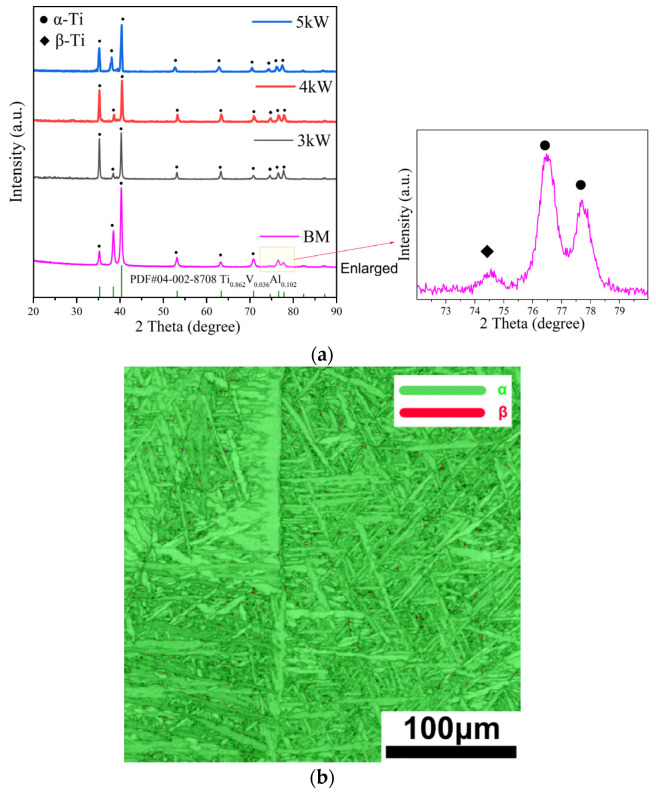
Phase composition characterization results of the FZ in a Ti-4Al-2V joint formed via underwater wet welding using varying laser power: (**a**) the XRD pattern, (**b**) the EBSD characterization results of a 3 kW joint.

Figure 6a shows the XRD patterns of the FZ of the Ti-4Al-2V joints. The FZ in all three joints, simultaneously contains α-Ti and β-Ti phases, but the content of the β-Ti phase is extremely low. As the laser power increases, the width of the half-maximum of the α phase increases, and the corresponding crystal plane of the 5 kW weld joint has a lower diffraction angle. According to the Bragg equation, it can be inferred that its lattice constant is larger, indicating that the lattice distortion of the α phase is severe under higher laser power. As commonly understood, this is due to the insufficient diffusion of elements in titanium alloys during high-speed cooling, resulting in excessive solid solution elements in the α phase and the lattice distortion. In addition, the diffraction intensity of the β phase decreases with increasing laser power, indicating a greater heat input leading to more sufficient β → α transformation. Figure 6b shows the phase composition of the 3 kW FZ joint using EBSD. The α-Ti and β-Ti content account for about 99.5% and 0.5%, respectively, which is consistent with the XRD results.

**Figure 7:**
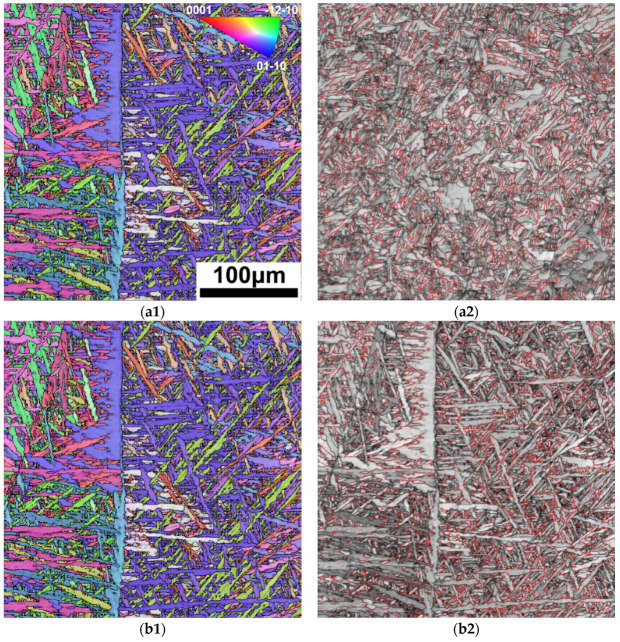
IPF and boundary distribution of FZ and HAZ in 3 kW joint: (**a1**) IPF figure of HAZ; (**a2**) 60°/<11–20> boundary distribution of HAZ; (**b1**) IPF diagram of FZ; (**b2**) 60°/<11–20> boundary distribution of FZ.

Figure 7 shows the Inverse pole figure (IPF) of the FZ and HAZ in the 3 kW joint, as well as the grain boundary distribution in corresponding areas. As shown in Figure 7(a1), the α phases show lath-like morphology with a small aspect ratio in the HAZ. In the represented field of view, the overall distribution of the α phase with different orientations is relatively uniform, but there are cluster features in specific areas. As shown by the red line in Figure 7(a2), 40% of the α-phase interface is 60°/<11–20>, far exceeding the random theoretical value—18.2%—indicating that there is a clear variant selection during the α phase precipitation, and Type-I clusters are mainly present [10].

Compared with the HAZ, the FZ, as shown in Figure 7(b1), is composed of an α lath with a larger ratio of length to width, and the size of the α variant cluster is significantly larger, which is consistent with its high cooling rate. As shown by the red line in Figure 7(b2), a 60°/<11–20> type interface accounts for more than 47%, indicating that Type-I clusters are also the main converge mode of FZ variants. The formation of Type-I clusters can minimize the interface energy during variant precipitation, which has been observed in our previous studies on SLM-formed and laser welding Ti-4Al-2V alloys. Similar clusters have also been characterized in commercial pure titanium, Ti-6Al-4V, and other titanium alloys [28,29,30,31].

### 3.4. Formation and Propagation of Cracks

**Figure 8:**
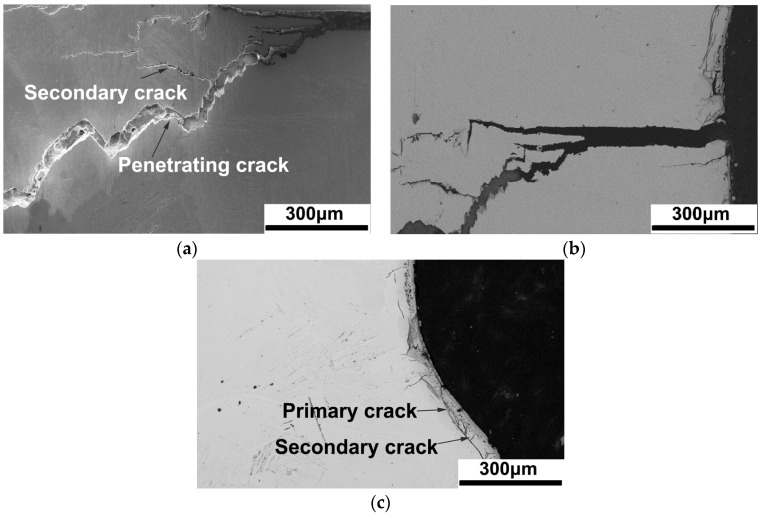
Morphology of cracks in the 4 kW joint: (**a**) the middle of the penetrating crack, (**b**) the surface of the penetrating crack, (**c**) the surface away from the penetrating crack.

As shown in Figure 2 and Figure 3, both 4 kW and 5 kW laser power resulted in penetrating cracks in the FZ. Further characterization of the crack morphology is shown in Figure 8. The SE2 image in Figure 8a shows that the crack penetrates the FZ in the 4 kW joint. It is indicated that multiple cracks show transgranular features, passing through the original β grain and breaking up the α laths, indicating that the cracks were generated and propagated after the formation of the α phase. By characterizing the positions near and far away from the penetrating crack, small cracks can be observed, as shown in Figure 8b,c. In Figure 8b, there are multiple parallel small cracks near the penetrating crack, and their propagation direction is from the oxide layer towards the interior of the matrix. In the middle of the FZ, in addition to a small crack, a slender crack perpendicular to the penetrating crack and parallel to the surface of the FZ can also be seen in Figure 8c.

The formation and propagation of weld cracks can be drawn as follows: (1) once the high-power laser is applied to the titanium alloy, a molten pool is formed, meanwhile, welding heat acts on water producing O_2_, and O_2_ reacts with the molten pool to form oxides; and (2) during the solidification process, the cooling rate is extremely high in the underwater environment, which leads to large internal stress inside the FZ, and this internal stress gradually accumulates during the L → β → α phase transformation. As the internal stress gradually increases, cracks firstly appear in the loose oxide layer, which extends towards the matrix. (3) After one of the cracks preferentially expands, the internal stress is released, and other cracks stop expanding. Therefore, the depth of other primary and secondary cracks, as shown in Figure 8, is much smaller than that of the penetrating cracks. (4) In the center of the FZ, due to the contraction of the molten pool, internal stress is directed towards the center of the molten pool, and cracks here preferentially propagate parallel to the surface of the FZ.

After the formation of cracks, the quality of the weld formation significantly decreases. The mechanical properties of the weld will rapidly deteriorate, such as in the tensile property of the 5 kW joint in Section 3.5, due to the formation of welding cracks. Furthermore, when cracks propagate towards the matrix, not only does the weld formation deteriorate further, but it will also lead to the complete disappearance of the weld’s tensile performance.

### 3.5. Tensile Property and Vickers Hardness

**Figure 9:**
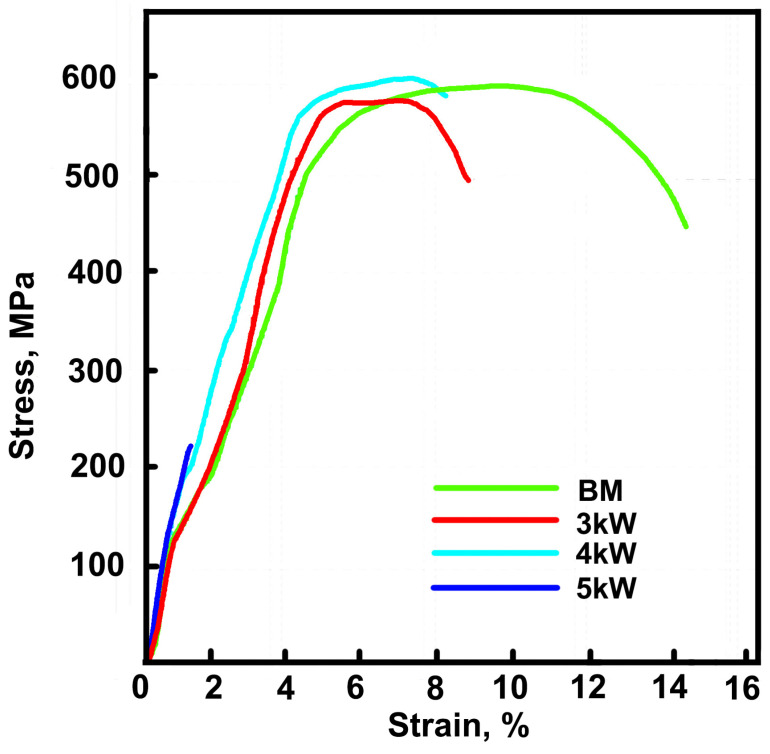
The room temperature tensile curves of the different joints and the BM.

The room temperature tensile curves of different joints, as well as the BM, are shown in Figure 9, and the corresponding rupture stress and fracture elongation are listed in Table 1. It is important to point out that all of the samples avoided weld cracks. The deformation capability of the three joints is deteriorated, and the strength of the 3 kW and 4 kW joints equivalent compared with the BM. The 4 kW joint shows the best comprehensive performance with a tensile strength of 604 MPa and fracture elongation of 7.14%, respectively. The performance of the 3 kW joint is accepted, but the 5 kW joint shows obvious brittleness without plasticity. The yielding stress is difficult to detect in the tensile curves, because there is no obvious yield plateau and the curves are not smooth enough. However, different joints exhibit similar tensile deformation behavior before fracture, according to the curves shown in Figure 9.

**Figure 10:**
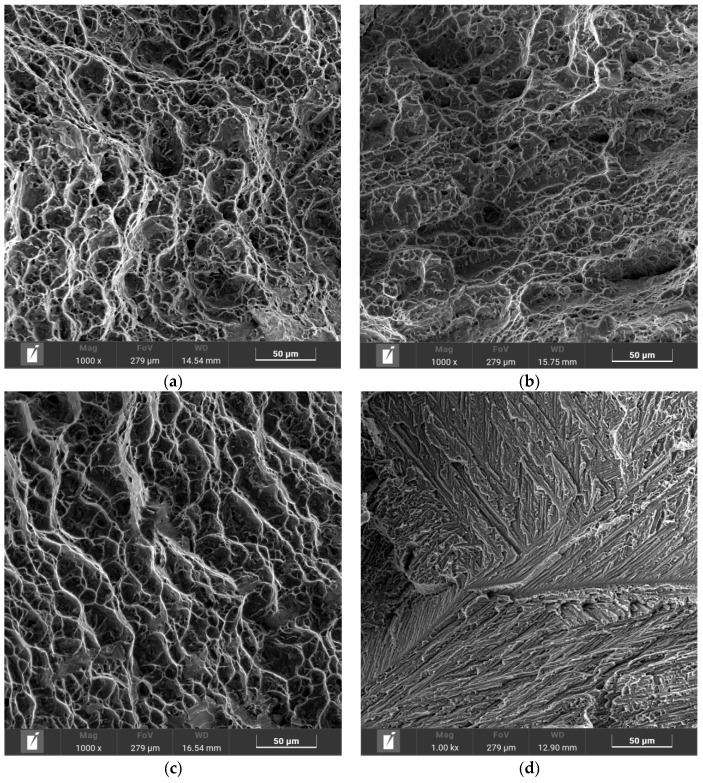
Tensile fracture morphology of BM and three joints: (**a**) BM, (**b**) 3 kW joint, (**c**) 4 kW joint, (**d**) 5 kW joint.

Figure 10 shows the tensile fracture morphology of the BM and the three joints. In the morphology of the BM, 3 kW and 4 kW joints, a typical ductile fracture occurs with obvious ductile dimples. The fracture dimples of the 3 kW and 4 kW joints is smaller and shallower compared with the BM, which is in line with the lower fracture elongation. There are obvious tearing edges in the fracture surface of the 5 kW joint, which is a typical brittle fracture.

When the welding quality is excellent, the strength of a titanium alloy weld joint is usually slightly higher than that of the BM, but the elongation is lower [12,18]. In this study, due to the influence of internal cracks, oxidation, and other defects in the weld, the strength of the weld did not exceed that of the base material, and some welds experienced brittle fractures, which is consistent with the microstructure and crack characterization results in Section 3.1, Section 3.2, Section 3.3 and Section 3.4

**Figure 11:**
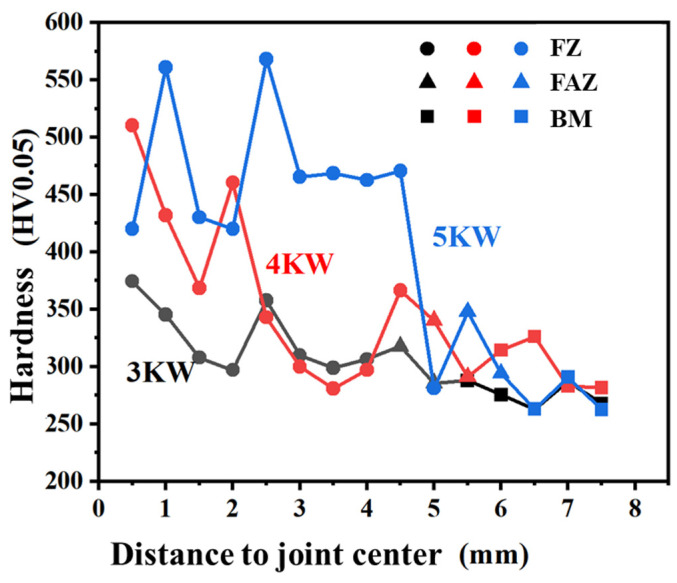
Micro-Vickers hardness of different welded joints.

The micro-Vickers hardness curves of the 3 kW, 4 kW, and 5 kW joints are shown in Figure 11. The hardness of the FZ and HAZ of the three joints is higher than 280 HV0.05 of the BM. The hardness of the 5 kW joint is the highest, mostly above 460 HV0.05, and can reach up to 568 HV0.05. The hardness of the 3 kW joint is the lowest, mostly around 300 HV0.05, with the highest being 374 HV0.05. The micro-Vickers hardness is related to the phase composition and compositional segregation in alloys. In the titanium weld joints, the needle-like α phase formed in the fusion zone material during rapid cooling is generally higher in hardness due to the presence of more solid solution elements inside. This is consistent with our previous research on argon arc welding and laser welding of the same alloy [12].

## 4. Conclusions

In this study, underwater wet laser welding of Ti-4Al-2V alloy was conducted using 3 kW, 4 kW, and 5 kW laser power at a depth of 4 mm, and the microstructure and properties were characterized. The main conclusions are as follows:

(1) In all the three joints, typical FZ and HAZ can be distinguished. The 3 kW joint has small cracks, but penetrating cracks can be seen in both the 4 kW and 5 kW joints. The cracks are located in the FZ, and their propagation direction is basically perpendicular to the surface of the FZ. The FZ is dominated by elongated α martensite, and the HAZ is full of lath α phase.
(2) The oxide layer can be detected on the surface of the FZ in all three joints, and its thickness gradually decreases from the middle of the FZ to its edge. The oxide consists of two layers, namely, an outer layer of TiO_2_ with a 10 μm thickness, and an inner layer of Al_2_O_3_ with a 1 μm thickness. Compared with the FZ, the surface of the HAZ and the BM have almost no oxide layer.
(3) Because of the internal stress accumulated from solidification and the solid state phase transformation during welding, a crack begins to generate and propagate perpendicular to the direction of the internal stress. A penetrating crack formed combining with small cracks.
(4) The tensile strength of the 3 kW and 4 kW joints is comparable to that of the BM, which is about 600 MPa, while the strength of the 5 kW joint significantly decreases to 228 MPa. The fracture elongation of the three joints is all significantly lower than that of the BM. Brittle fracture occurred in the 5 kW joint, which is related to the excessive proportion of internal cracks in the joint. The micro-Vickers hardness of the FZ in all three joints is higher than that of the BM, and significantly increases with the increase in laser power.

## Figures and Tables

**Table 1:** The room temperature tensile properties of the 3 kW, 4 kW, and 5 kW joints, and the BM.

	BM	3 kW Joint	4 kW Joint	5 kW Joint
Tensile strength, MPa	593 ± 3	583 ± 2	602 ± 2	220 ± 8
Fracture elongation, %	16.4 ± 2.2	7.7 ± 1.1	7.6 ± 0.6	0

## Data Availability

Data are contained within the article.
